# Efficacy of cognitive-behavioral therapy in patients with bipolar disorder: A meta-analysis of randomized controlled trials

**DOI:** 10.1371/journal.pone.0176849

**Published:** 2017-05-04

**Authors:** Kai-Jo Chiang, Jui-Chen Tsai, Doresses Liu, Chueh-Ho Lin, Huei-Ling Chiu, Kuei-Ru Chou

**Affiliations:** 1 School of Nursing, College of Nursing, Taipei Medical University, Taipei, Taiwan; 2 Department of Nursing, Tri-Service General Hospital, National Defense Medical Center, Taipei, Taiwan; 3 Department of Nursing, Taipei Medical University-Shuang Ho Hospital, Taipei, Taiwan; 4 Department of Nursing, Wan Fang Hospital, Taipei Medical University, Taipei, Taiwan; 5 School of Gerontology Health Management, College of Nursing, Taipei Medical University, Taipei, Taiwan; 6 Master’s Program in Long-Term Care, College of Nursing, Taipei Medical University, Taipei, Taiwan; 7 Psychiatric Research Center, Taipei Medical University Hospital, Taipei, Taiwan; Universita Cattolica del Sacro Cuore Sede di Roma, ITALY

## Abstract

**Background:**

Although cognitive behavioral therapy (CBT) is considered a promising adjuvant to pharmacotherapy for treating bipolar disorder (BD), its efficacy is unproven. The present review and meta-analysis evaluated the treatment outcomes of patients with BD treated with CBT plus medication and compared these data with the outcomes of those who received standard care alone.

**Methods:**

Electronic searches from inception to July 31, 2016, were performed using PubMed, Medline OVID, Cochrane Library, EMBASE, CINAHL plus, and PsycINFO. In the extensive electronic literature search, keywords such as “bipolar disorder,” “manic-depressive psychosis,” “bipolar affective disorder,” “bipolar depression,” “cognitive therapy,” “cognitive-behavioral therapy,” and “psychotherapy” were transformed into MeSH terms, and only randomized controlled trials (RCTs) were included. The pooled odds ratios (ORs) of relapse rates and Hedges’s g, along with 95% confidence intervals (CIs), for the mean differences in the levels of depression, mania, and psychosocial functioning were calculated. Further subgroup analyses were conducted according to the characteristics of the CBT approaches, patients, and therapists, if the data were available.

**Result:**

A total of 19 RCTs comprising 1384 patients with type I or II BD were enrolled in our systematic review and meta-analysis. The main analysis revealed that CBT could lower the relapse rate (pooled OR = 0.506; 95% CI = 0.278 −0.921) and improve depressive symptoms (g = −0.494; 95% CI = −0.963 to −0.026), mania severity (g = −0.581; 95% CI = −1.127 to −0.035), and psychosocial functioning (g = 0.457; 95% CI = 0.106–0.809).

**Conclusions:**

CBT is effective in decreasing the relapse rate and improving depressive symptoms, mania severity, and psychosocial functioning, with a mild-to-moderate effect size. Subgroup analyses indicated that improvements in depression or mania are more potent with a CBT treatment duration of ≥90 min per session, and the relapse rate is much lower among patients with type I BD.

## Introduction

Bipolar disorder (BD) is a severe mental disease with a lifelong course and considerable morbidity and mortality. BD has a lifelong prevalence rate of 1%–1.5% and is characterized by recurrent episodes of mania, depression, or a mixture of both phases [1]. BD can cause impaired cognition [2], functional decline [3], poor health outcomes [4], and a high frequency of suicidal behavior [5]. The inter-personal relationships of patients with BD are also highly affected by the dramatic alternation of manic/hypomanic and depressive mood cycles. A BD cohort study with a relatively large sample size (n = 1469) demonstrated that 58% of patients with BD types I and II recovered, but approximately half of them experienced recurrence within 2 years [6]. In the United States, the direct and indirect costs of BD were estimated to be USD 151 billion in 2009 [7]. Millions of patients worldwide are affected by this severe mood illness, incurring costs of billions of USD for the years lived with disability [8].

Given the biological and hereditary underpinning of BD, pharmacotherapy is the first-line treatment. However, a growing body of literature suggests that combined pharmacotherapy and psychotherapy is more effective in treating patients with BD than is medication alone [9]. As an adjuvant therapy, psychotherapy helps patients with BD in improving their compliance, awareness, and coping skills for life events, which collectively results in an improved response to pharmacotherapy [10–13, 32]. Among the psychological therapies that are potential adjuncts to medications for patients with BD, cognitive-behavioral therapy (CBT) is a promising treatment option but has inconclusive findings [14].

In clinical settings, CBT is the non-pharmaceutical intervention of choice for patients with depression and anxiety, the core concept and treatment practice model were developed by Beck et al. more than 40 years previously [15, 16]. Randomized controlled trials (RCTs) published within the past 10 years have disclosed the potential benefits of CBT as an adjunct to mood stabilizers for preventing relapse, relieving symptoms, and enhancing drug adherence [9]. Currently, some meta-analyses have evaluated the efficacy of CBT for BD [17–23]. These studies have demonstrated that CBT has a small impact on clinical symptoms [17–19], but the evidence remains incomprehensive and inconclusive due to limited data. In a meta-analysis, Ye et al described the short-term efficacy of CBT in lowering the relapse rate of BD [19]. In our study, an in-depth subgroup analysis of the meta-analyses on this topic was conducted to provide insights for psychiatrists and psychologists. Accordingly, we performed a meta-analysis, as well as extensive searches of multiple databases and further subgroup analysis, to determine the efficacy of CBT in improving depressive symptoms, mania severity, relapse rates and social functioning.

## Materials and methods

### Reporting standards for meta-analyses

This meta-analysis was performed according to the Preferred Reporting Items for Systematic Reviews and Meta-analysis (PRISMA) statement for the meta-analyses of RCTs. The PRISMA checklist(S1 Checklist) is provided as Supplementary Material.

### Search strategy for systematic literature reviews

Electronic searches from the date of inception to July 31, 2016 were performed using PubMed, Medline OVID, the Cochrane Library, EMBASE, CINAHL Plus, and PsycINFO. To identify specific and relevant studies, we developed a search strategy based on the patient population (BD), treatment (CBT), and study design (RCT; S1 Table). In extensive electronic literature searches, keywords such as “bipolar disorder,” “manic-depressive psychosis,” “bipolar affective disorder,” “bipolar depression,” “cognitive therapy,” “cognitive-behavioral therapy,” and “psychotherapy” were transformed into exploded MeSH terms. The references from selected articles were also accessed for eligibility in the review process. All the candidate articles were evaluated by two independent reviewers through systematic approaches involving the inclusion and exclusion criteria.

### Selection criteria

In this systematic review, eligible studies fulfilled the following inclusion criteria: (1) RCT, (2) patients with BD aged ≥18 years, (3) presence of two study groups: a comparison group receiving the usual interventions and a CBT group receiving CBT plus the intervention given to the control group, and (4) availability of at least one relevant outcome such as changes in the relapse rate, depressive symptoms, mania severity, and psychosocial functioning. The depressive symptoms were assessed using the Hamilton Rating Scale for Depression (HRSD), Beck Hopelessness Scale (BHS), Beck Depression Inventory (BDI), or Montgomery–Asberg Depression Rating Scale (MADRS); the mania severity was assessed using the Mania Rating Scale (MRS) or Young Mania Rating Scale (YMRS); and the level of psychosocial functioning was assessed using the Global Assessment of Functioning (GAF), Dysfunctional Attitude scale (DAS), or Social Performance Scale (SPS).

The exclusion criteria were as follows: (1) no relevant data were available for further meta-analysis and (2) article types other than RCTs, such as comments, letters, and reviews. In addition, for duplicated publications with the same study participants, only those studies with the most relevant and comprehensive data were considered, and the other studies were discarded. Furthermore, during the selection process for systematic reviews, we verified whether CBT or the relevant variants were included in the psychological interventions of each study. However, some studies used psychological therapies based on CBT or CBT-modified programs. After careful discussion, we included such studies because their core psychological intervention was CBT.

### Data extraction, data verification, and quality assessment

Data extraction was performed by two independent reviewers who used a specific work sheet designated before the literature search. A consensus meeting was held with a third researcher to resolve disparities between the two reviewers. Data extraction was conducted from full-text versions of the RCTs, where available. A quality-control process for the data extraction was undertaken by another researcher to verify all the extracted data against the original sources. The data regarding basic characteristics and outcome measures, including the study identity (first author plus publication year), country, study design, number of study participants, mean or median age, gender, intervention characteristics, and all relevant outcomes, were extracted for all studies. Quality assessment of the selected RCTs was conducted according to Cochrane Collaboration’s tool for assessing the risk of bias in randomized trials [24]. If the study populations were duplicated, the most updated findings or most comprehensive outcome measures were chosen. Some of the follow-up studies of the original RCTs focused on cost-effectiveness and could not be included in this meta-analysis.

### Statistical analysis

All data analyses were performed using Comprehensive Meta-Analysis, Version 3.3 (Biostat Inc., Englewood, NJ, USA). The efficacy of CBT in lowering the relapse rate was evaluated from the overall odds ratio (OR). The average changes in the scores for depressive symptoms, mania severity, and psychosocial functioning were calculated from baseline to the study end. Furthermore, Hedges’s g was used to determine the effect size of continuous outcomes, with g values of 0.2–0.4, 0.5–0.7, and ≥0.8 representing small, moderate, and large effect sizes, respectively. The significance of the ORs and Hedges’s g was determined using a Z test. A two-tailed P <0.05 was considered statistically significant. The heterogeneity among RCTs was determined using the Cochran’s Q test and I^2^ statistic, with I^2^ values of 75%, 50%, 25%, and 0% indicating high, moderate, low, and no heterogeneity, respectively. Data from individual RCTs were summarized using a random-effects model for obtaining more statistically conservative estimates compared with those obtained using a fixed-effects model. In addition, further subgroup analysis was conducted according to the characteristics of CBT approaches, patients, and therapists, if the data were available for assessing the impact of different characteristics on the efficacy of CBT in treating BD. Sensitivity analyses were performed using the leave-one-out approach to elevate the robustness of the pooled estimates. Publication bias was evaluated using a funnel plot with Egger’s test.

## Results

### Characteristics of the included studies

**Fig 1:**
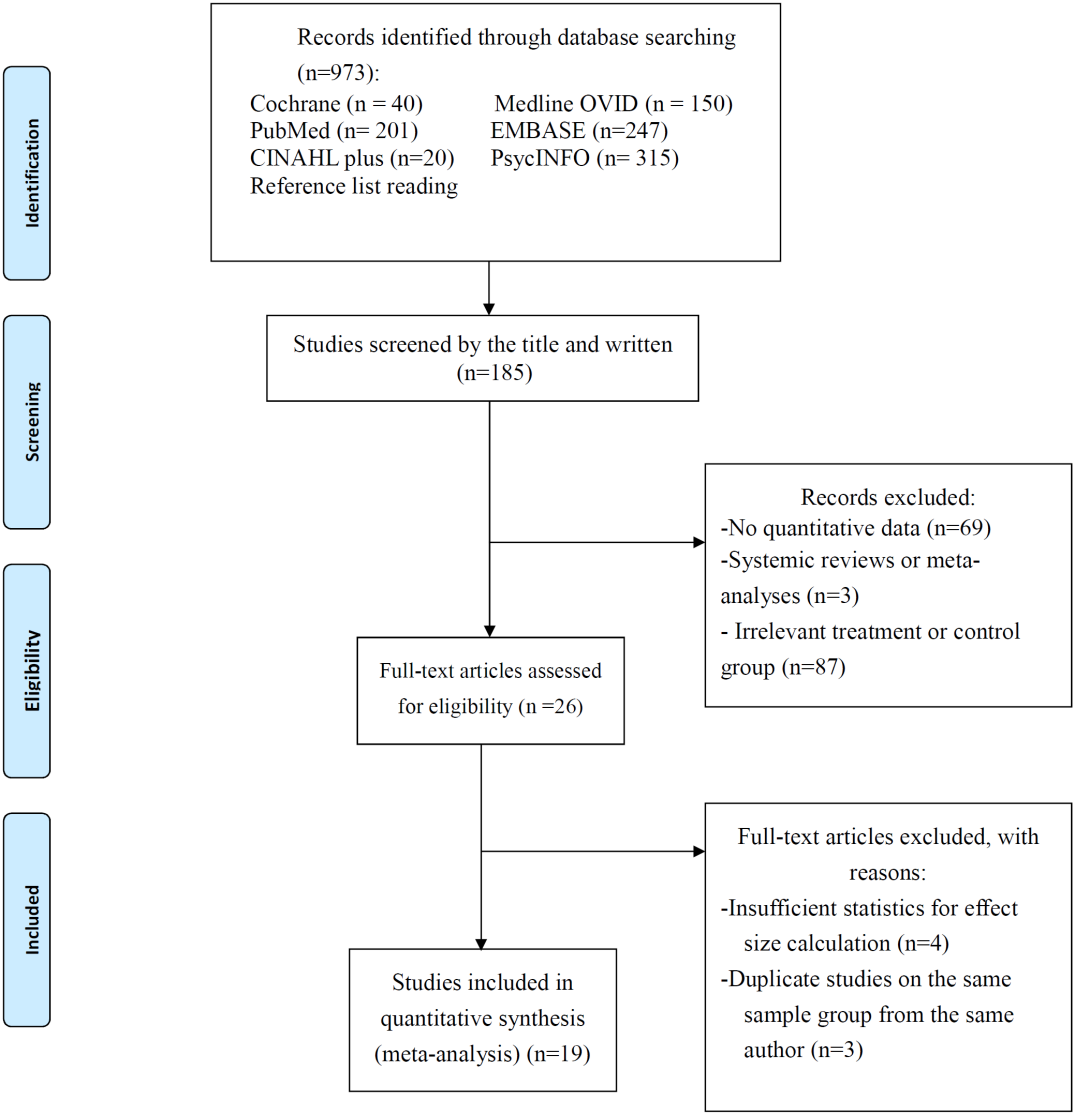
PRISMA 2009 flow diagram.

Fig 1 depicts the entire literature review process. Initially, 973 research reports were identified. Through independent reviews based on the inclusion/exclusion criteria, 19 RCTs evaluating the efficacy of CBT for patients with BD were included in this meta-analysis [25–43]; these studies comprised a total of 716 patients with CBT and 668 controls. Of the 19 RCTs, three included only patients with BD I, and other studies included patients with BD I or II. The mean age of the patients at enrollment ranged from 34.7 to 44 years. Most of these RCTs (n = 10) used individual-based CBT, and other studies (n = 9) used group-based CBT. The number of CBT sessions ranged from 8 to 30, and the duration of each session ranged from 45 to 120 min. All the included RCTs were published between 2000 and 2015. Other details are summarized in Table 1.

**Table 1:** Characteristics of cognitive-behavioral therapy for patients with bipolar disorder in the randomized controlled trials included in the meta-analysis (N = 19).

Study	Country	Intervention (experimental/control)	Design	Sample size (patients)	Intervention characterization	Outcome	Study quality/Cochrane tool
Lam et al. (2000) [25]	UK	CBT/TAU	RCT	• Total N: 25	• Number of sessions treated: 12–20	• Relapse rate (episodes)	7/
				• Complete N (T/C): 12/11	• Treatment length (min): -	• Depression level (BDI, BHS, HDRS)	AA: low
				• Mean age (years):39.0	• Each group size (people): individual	• Mania severity (MRS)	AC: unclear
				• Gender (M/F): 12/13	• CBT therapist: certified professional	• Function (SPS)	BAO: low
				• Bipolar I: 25		• Assessed time points (months): 6, 12	IO: low
							SRO: low
Scott et al. (2001) [26]	UK	CT/WLC	RCT	• Total N: 42	• Number of sessions treated: 25	• Relapse rate (episodes)	7/
				• Complete N (T/C): 21/21	• Treatment length (min): 45	• Depression level (BDI)	AA: low
				• Mean age (years):38.8	• Each group size (people): individual	• Symptom (ISS)	AC: unclear
				• Gender (M/F): 17/25	• CBT therapist: certified professional	• Function (GAF)	BAO: unclear
				• Bipolar I: 34 • Bipolar II: 8		• Assessed time points (months): 6, 12, 18	IO: low
							SRO: low
Lam et al. (2003) [27]	UK	CT/TAU	RCT	• Total N: 103	• Number of sessions treated: 12–18	• Relapse rate (episodes)	6/
				• Complete N (T/C): 43/44	• Treatment length (min): -	• Depression level (BDI, HDRS)	AA: low
				• Mean age (years):44.0	• Each group size (people): individual	• Mania severity (MRS)	AC: unclear
				• Gender (M/F): 45/58	• CBT therapist: certified professional	• Function (SPS, DAS)	BAO: unclear
				• Bipolar I: 103		• Assessed time points (months): 6, 12, 18	IO: low
							SRO: low
Ball et al. (2006) [28]	Australia	CT/TAU	RCT	• Total N: 52	• Number of sessions treated: 20	• Relapse rate (episodes)	7/
				• Complete N (T/C): 25/27	• Treatment length (min): 60	• Depression level (BDI, BHS, HDRS, MADRS)	AA: low
				• Mean age (years):42.0	• Each group size (people): individual	• Mania severity (YMRS)	AC: unclear
				• Gender (M/F): 22/30	• CBT therapist: certified professional	• Function (GAF, SAS, SPS, DAS)	BAO: low
				• Bipolar I or II: 52		• Assessed time points (months): 3, 6, 9, 12	IO: low
							SRO: low
Scott et al. (2006) [29]	UK	CBT/SC	RCT	• Total N: 253	• Number of sessions treated: 22	• Recurrence rate (episodes)	8/
				• Complete N (T/C): 127/126	• Treatment length (min): -	• Depression level (LIFE-II score for depression)	AA: low
				• Mean age (years):41.2	• Each group size (people): individual	• Mania severity (LIFE-II score for mania)	AC: unclear
				• Gender (M/F): 89/164	• CBT therapist: certified professional	• Assessed time points (months): 6, 12, 18	BAO: low
				• Bipolar I: 238 • Bipolar II: 15			IO: low
				• Recurrent Bipolar			SRO: low
Miklowitz et al. (2007) [30]	USA	IPT/CC	RCT	• Total N: 152	• Number of sessions treated: 30	• Depression level (MADRS)	8/
				• Complete N (T/C): 84/68	• Treatment length (min): 60	• Function (LIFE-RIFT)	AA: low
				• Mean age (years):41.1	• Each group size (people): individual	• Assessed time points (months): 3, 6, 9	AC: unclear
				• Gender (M/F): 64/88	• CBT therapist: unclear		BAO: low
				• Bipolar I: 105 • Bipolar II: 47			IO: low
							SRO: low
William et al. (2008) [31]	UK	MBCT/WLC	RCT	• Total N: 68	• Number of sessions treated: 8	• Depression level (BDI-II)	8/
				• Complete N (T/C): 28/27	• Treatment length (min): 120	• Anxiety level (BAI)	AA: low
				• Age (years):18–65	• Each group size (people): 12–15	• Assessed time points (months): post-treatment	AC: unclear
				• Gender (M/F): 12/13	• CBT therapist: certified professional		BAO: low
				• Unipolar: 51 • Bipolar: 17			IO: low
							SRO: low
Zaretsky et al. (2008) [32]	Canada	CBT/PE	RCT	• Total N: 79	• Number of sessions treated: 14	• Depression level (HDRS)	6/
				• Complete N (T/C): 29/24	• Treatment length (min): -	• Mania severity (CARS-M)	AA: low
				• Mean age (years): 40.7	• Each group size (people): individual	• Function (SPS, DAS)	AC: unclear
				• Gender (M/F): unclear	• CBT therapist: certified professional	• Assessed time points (weeks): 2, 6, 12	BAO: unclear
				• Bipolar I: 52 • Bipolar II: 27			IO: low
							SRO: low
Perlick et al. (2010) [33]	USA	FF-CBT/HE	RCT	• Total N: 46	• Number of sessions treated: 12–15	• Depression level (HDRS)	8/
				• Complete N (T/C): 22/18	• Treatment length (min): 45	• Mania severity (YMRS)	AA: low
				• Mean age (years):34.7	• Each group size (people): family-based	• Assessed time points (months): post-treatment	AC: unclear
				• Gender (M/F): 15/25	• CBT therapist: unclear		BAO: low
				• Bipolar I: 40 • Bipolar II: 6			IO: low
							SRO: low
Costa et al. (2011) [34]	Brazil	CBGT/TAU	RCT	• Total N: 41	• Number of sessions treated: 14	• Depression level (BDI, BHS)	7/
				• Complete N (T/C): 27/12	• Treatment length (min): 120	• Mania severity (YMRS)	AA: low
				• Mean age (years):40.5	• Each group size (people): group	• Anxiety level (BAI)	AC: unclear
				• Gender (M/F): 12/25	• CBT therapist: certified professional	• Assessed time points (weeks): 7, 14	BAO: low
				• Bipolar I: 35 • Bipolar II: 6			IO: low
							SRO: low
Gomes et al. (2011) [35]	Brazil	CBGT/TAU	RCT	• Total N: 50	• Number of sessions treated: 18	• Relapse rate (episodes)	7/
				• Complete N (T/C): 25/22	• Treatment length (min): 90	• Assessed time points (months): post-treatment	AA: low
				• Median age (years):38.0	• Each group size (people): 4.4		AC: unclear
				• Gender (M/F): 12/38	• CBT therapist: certified professional		BAO: low
				• Bipolar I: 38 • Bipolar II: 12			IO: low
							SRO: low
Meyer et al. (2012) [36]	Germany	CBT/ST	RCT	• Total N: 76	• Number of sessions treated: 20	• Recurrence rate (episodes)	8/
				• Complete N (T/C): 38/38	• Treatment length (min): 50–60	• Depression level (BDI, BHS)	AA: low
				• Mean age (years):44.0	• Each group size (people): individual	• Mania severity (SRMI)	AC: low
				• Gender (M/F): 38/38	• CBT therapist: certified professional	• Function (GAS)	BAO: unclear
				• Bipolar I: 38 • Bipolar II: 38		• Assessed time points (months): post-treatment	IO: low
							SRO: low
Harvey et al. (2015) [37]	USA	CBT/PE	RCT	• Total N: 58	• Number of sessions treated: 8	• Relapse rate (episodes)	8/
				• Complete N (T/C): 30/28	• Treatment length (min): 50–60	• Insomnia (ISI, SD-SE, PSQI)	AA: low
				• Mean age (years):36.6	• Each group size (people): individual	• Depression level (IDS-C)	AC: unclear
				• Gender (M/F): 22/36	• CBT therapist: certified professional	• Mania severity (YMRS)	BAO: low
				• Bipolar I: 58		• Function (SDS)	IO: low
						• Assessed time points (months): post-treatment, 6 month follow-up	SRO: low
Colom et al. (2003) [38]	Spain	GPE/SC	RCT	• Total N: 120	• Number of sessions treated: 21	• Recurrence rate (episodes)	8/
				• Complete N (T/C): 60/60	• Treatment length (min): 90	• Hospitalization	AA: low
				• Mean age at onset (years): 22.8	• Each group size (people): 8 to 12	• Assessed time points (months): 6, 12, 18, 24-month follow-up	AC: unclear
				• Gender (M/F): 44/76	• CBT therapist: certified professional		BAO: low
				• Bipolar I: 100 • Bipolar II: 20			IO: low
							SRO: low
González-Isasi et al. (2010) [39]	Spain	CT/SC	RCT	• Total N: 40	• Number of sessions treated: 20	• Relapse	8/
				• Complete N (T/C): 20/20	• Treatment length (min): 90	• Depression level (BDI)	AA: low
				• Mean age (years): 41.3	• Each group size (people): 10	• Mania severity (YMRS)	AC: low
				• Gender (M/F): 21/19	• CBT therapist: certified professional	• Anxiety level (STAI-S)	BAO: unclear
				• Bipolar I/II: 40 • Refractory Bipolar		• Inadaption scale	IO: low
						• Assessed time points (months): post-treatment, 6, 12 months follow-up	SRO: low
Weiss et al. (2007) [40]	USA	IGT/SC	RCT	• Total N: 62	• Number of sessions treated: 20	• Substance use	8/
				• Complete N (T/C): 31/31	• Treatment length (min): 60	• Depression level (HAM-D)	AA: low
				• Mean age (years):41.9	• Each group size (people): -	• Mania severity (YMRS)	AC: unclear
				• Gender (M/F): 30/32	• CBT therapist: certified professional	• Assessed time points (months): post-treatment, 3, 5, 8 months follow-up	BAO: low
				• Bipolar I: 40 • Bipolar II: 22			IO: low
				• SUD			SRO: low
Weiss et al. (2009) [41]	USA	IGT/GDC	RCT	• Total N: 61	• Number of sessions treated: 12	• Substance use	8/
				• Complete N (T/C): 31/30	• Treatment length (min): 60	• Relpase rate (episode)	AA: low
				• Mean age (years): 38.3	• Each group size (people): -	• Addiction (ASI)	AC: low
				• Gender (M/F): 36/25	• CBT therapist: certified professional	• Depression level (HDRS)	BAO: unclear
				• Bipolar I: 48 • Bipolar II: 9		• Mania severity (YMRS)	IO: low
				• BD, NOS: 4 • SUD		• Assessed time points (months): post-treatment, 3, 6 months of follow-up	SRO: low
Perich et al. (2013) [42]	Australia	MBCT/TAU	RCT	• Total N: 95	• Number of sessions treated: 8	• Recurrence rate (episodes)	8/
				• Complete N (T/C): 48/47	• Treatment length (min): 120–150	• Depression level (MADRS, DASS)	AA: low
				• Mean age (years):-	• Each group size (people): individual	• Mania severity (YMRS)	AC: unclear
				• Gender (M/F): 33/62	• CBT therapist: certified professional	• Anxiety level (STAI)	BAO: low
				• Bipolar I: 59 • Bipolar II: 35		• Assessed time points (months): post-treatment, 3, 6, 9, and 12 months of follow-up	IO: low
				• Bipolar NOS: 1			SRO: low
González-Isasi et al. (2010) [43]	Spain	CBT/SC	RCT	• Total N: 20	• Number of sessions treated: 13	• Relapse	8/
				• Complete N (T/C): 20/20	• Treatment length (min): 90	• Depression level (BDI)	AA: low
				• Mean age (years): 38.5	• Each group size (people): 10	• Mania severity (YMRS)	AC: low
				• Gender (M/F): 6/14	• CBT therapist: certified professional	• Function (GAF)	BAO: unclear
				• Bipolar I/II: 40 • Refractory Bipolar		• Assessed time points (months): post-treatment, 6, 12 months follow-up	IO: low
							SRO: low

CBT: cognitive-behavioral therapy, TAU: treatment as usual, WLC: waiting list control, MM: medication monitoring, SC: standard care, SUD: substance use disorder, IPT: intensive psychosocial treatment, CC: collaborative care, MBCT: mindfulness-based cognitive therapy, PE: psychoeducation, FF-CBT: family-focused, cognitive behavioral therapy, treatment-health promoting intervention, HE: health education, ST: supportive therapy

### Primary outcome: Depressive symptoms and mania severity

**Fig 2:**
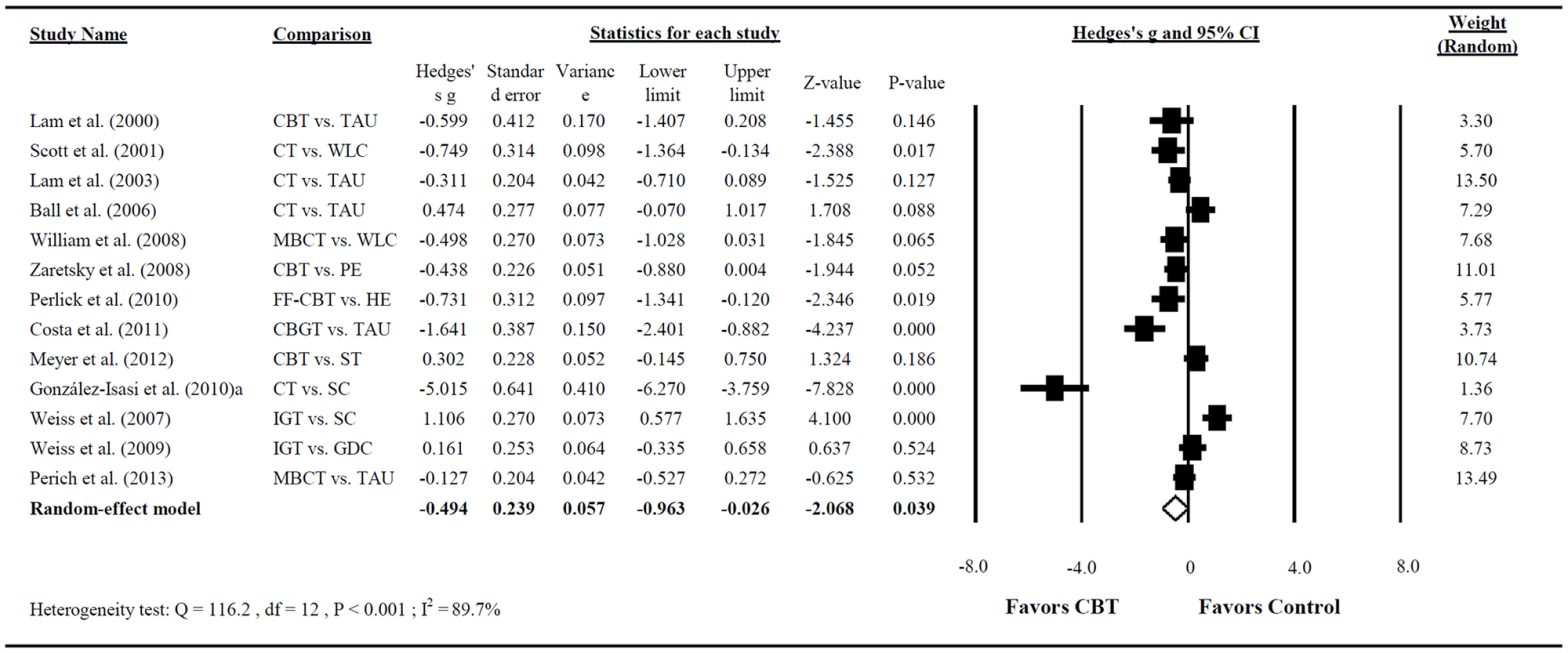
Forest plot of the meta-analysis for reducing depressive symptoms in BD among patients treated with CBT compared with controls (n = 13 studies).

In 13 RCTs reporting treatment outcomes concerning depression [25–28, 31–34, 36, 39–42], which employed the BDI, BHS, HRSD, and MADRS, the pooled effect size indicated that patients who underwent CBT exhibited a more favorable response in terms of decreased depression levels compared with those treated as usual (Hedges’s g = −0.494; 95% CI = −0.963 to −0.026; P = 0.039, with a moderate effect size; Fig 2). Large heterogeneity was observed in this analysis (Q = 116.179; P < 0.001; I^2^ = 89.671%).

**Fig 3:**
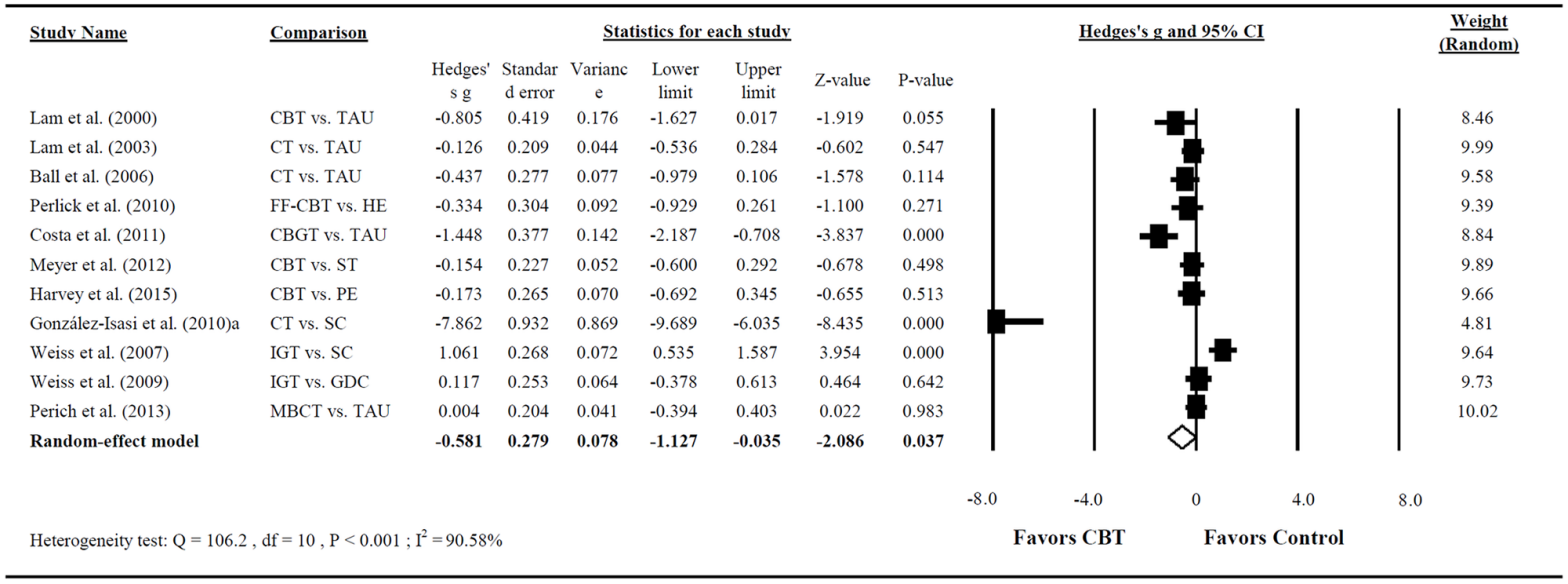
Forest plot of the meta-analysis for reducing mania severity in BD among patients treated with CBT compared with controls (n = 11 studies).

In 11 RCTs reporting the treatment efficacy with respect to mania [25, 27, 28, 33, 34, 36, 37, 39–42], which employed the MRS and YMRS, the pooled effect size indicated that CBT significantly reduced the severity of mania in patients with BD (Hedges’s g = −0.581; 95% CI = −1.127 to −0.035; P = 0.037, with a moderate effect size; Fig 3). Large heterogeneity was noted in this analysis (Q = 106.210; P < 0.001; I^2^ = 90.585%).

### Secondary outcomes: Relapse rate and psychosocial functioning

**Fig 4:**
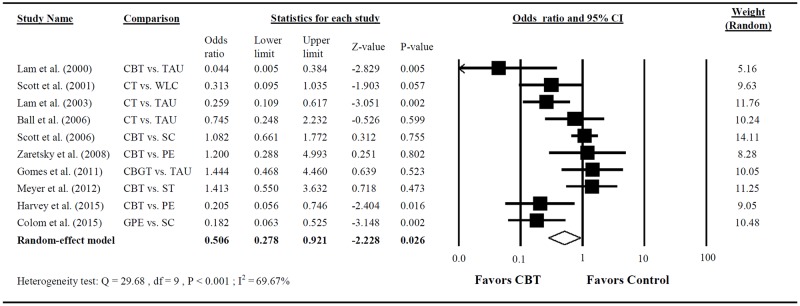
Forest plot of the meta-analysis for lowering the relapse rate of BD among patients treated with CBT compared with controls (n = 10 studies).

A total of 10 RCTs provided adequate statisticsal data for calculating the relapse rate [25–29, 32, 35–38]. The pooled OR indicated that compared with the control group, patients with CBT had significantly lower relapse rates at follow-up (Fig 4; pooled OR = 0.506; 95% CI = 0.278–0.921; P = 0.026). Large heterogeneity was observed in this analysis (Q = 29.676; P < 0.001; I^2^ = 69.672%).

**Fig 5:**
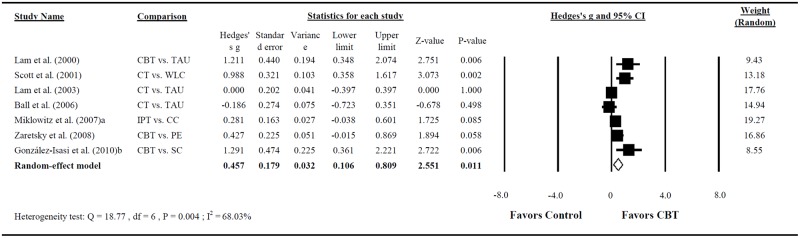
Forest plot of the meta-analysis for improving psychosocial functioning in BD among patients treated with CBT compared with controls (n = 7 studies).

In addition, our meta-analysis revealed the potential clinical benefits of CBT for improving psychosocial functioning (assessed through GAF, SPS, or DAS), according to the pooled findings of seven RCTs [25–28, 30, 32, 43] (Hedges’s g = 0.457; 95% CI = 0.106–0.809; P = 0.011, with a moderate effect size; Fig 5). Large heterogeneity was noted in this analysis (Q = 18.769; P < 0.001; I^2^ = 68.032%).

In summary, the overall effect size of CBT for patients with BD is shown in Table 2.

**Table 2:** Overall effect size of cognitive behavioral therapy for patients with bipolar disorder.

		Effect size	95% CI		Null hypothesis (two-sided)		Homogeneity			
Outcomes	Sample size (studies)		Lower	Upper	Z value	P value	Q value	P value	I^2^	τ^2^
• Depression	13	−0.494^a^	−0.963	−0.026	−2.068	0.039	116.179	<0.001	89.671	0.644
• Mania	11	−0.581^a^	−1.127	−0.035	−2.086	0.037	106.210	<0.001	90.585	0.734
• Relapse rate	10	0.506^b^	0.278	0.921	−2.228	0.026	29.676	<0.001	69.672	0.599
• Psychosocial functioning	7	0.457^a^	0.106	0.809	2.551	0.011	18.769	<0.001	68.032	0.141

CI, confidence interval

^a^ Hedges’ g

^b^ Odds ratio

### Sensitivity analysis

S1 Fig displays the effect of removing every single study on the overall estimate. In the random-effects model, the pooled ORs for the relapse rate did not exhibit marked dispersion, and similar findings for Hedges’s g were observed with regard to the level of depression, severity of mania, and level of psychosocial functioning. These findings consolidate the robustness of this meta-analysis, indicating that none of the studies would dominate the summarized results.

### Publication bias

S2 Fig illustrates the funnel plot for evaluating potential publication bias. The funnel plot shows symmetry, and the publication bias does not seem significant for the relapse rate, according to Egger’s test (P = 0.107).

### Subgroup analysis

Tables 3 and 4 present all the subgroup analyses for efficacy outcomes, according to the characteristics of the patients, therapists, and therapies. The subgroup analysis for the relapse rate revealed that studies assessing only patients with BD I reported a greater reduction in relapse rate after CBT than did studies assessing patients with BD I and II (Q_B_ = 7.889; P < 0.001). The subgroup analysis for the recovery of depressive symptoms indicated that the effect size was significantly larger for treatment durations of ≥90 min per session compared with that for treatment durations of <90 min per session (Q_B_ = 5.456; P = 0.019), and this effect with regard to treatment duration was also observed in the subgroup analysis for the reduction in mania severity (Q_B_ = 4.135; P = 0.042).

**Table 3:** Subgroup analysis of cognitive behavioral therapy for patients with bipolar disorder: Depression and mania.

	Depression (n = 13)					Mania (n = 11)				
	Sample size (studies)	Hedges’ g (95% CI)	P_A_	Q_B_	P_B_	Sample size (studies)	Hedges’ g (95% CI)	P_A_	Q_B_	P_B_
**Disease type**										
• Bipolar I	2	-0.367 (-0.725, -0.009)	0.044	0.211	0.646	3	-0.239 (-0.556, 0.078)	0.139	1.521	0.218
• Bipolar I/II	11	-0.523 (-1.081, 0.035)	0.066			8	-0.762 (-1.531, 0.006)	0.052		
**Therapist background**										
• Psychologists	12	-0.510 (-1.027, 0.006)	0.053	0.043	0.835	11	-0.581 (-1.127, -0.035)	0.037	0.000	1.000
• Nonpsychologists	1	-0.438 (-0.880, 0.004)	0.052			0				
**Therapy delivery**										
• Individual	7	-0.176 (-0.483, 0.131)	0.261	1.880	0.170	6	-0.186 (-0.383, 0.010)	0.063	2.491	0.114
• Group	6	-1.000 (-2.137, 0.137)	0.085			5	-1.407 (-2.910, 0.096)	0.067		
**Treatment sessions**										
• ≥12	8	-0.648 (-1.487, 0.192)	0.130	0.659	0.417	7	-0.951 (-1.893, -0.009)	0.048	3.080	0.079
• <12	2	-0.271 (-0.624, 0.083)	0.134			2	-0.062 (-0.378, 0.255)	0.703		
• undisclosed	3					2				
**Treatment frequency**										
• Otherwise	4	-0.510 (-1.228, 0.209)	0.164	0.000	0.994	5	-0.456 (-0.885, -0.027)	0.037	0.444	0.505
• Weekly	9	-0.506 (-1.135, 0.123)	0.115			6	-0.824 (-1.817, 0.170)	0.104		
**Treatment duration**										
• ≥90 min	4	-1.695 (-3.110, -0.280)	0.019	5.456	0.019	3	-2.902 (-5.683, -0.122)	0.041	4.135	0.042
• <90 min	6	0.110 (-0.429, 0.649)	0.689			6	0.017 (-0.410, 0.443)	0.940		
• undisclosed	3					2				

P_A_, subgroup effect on outcome variable; P_B_, heterogeneity among subgroups (moderator); CI, confidence interval

**Table 4:** Subgroup analysis of cognitive behavioral therapy for patients with bipolar disorder: Relapse rate and psychosocial functioning.

	Relapse rate (n = 10)					Psychosocial functioning (n = 7)				
	Sample size (studies)	Odds ratio (95% CI)	P_A_	Q_B_	P_B_	Sample size (studies)	Hedges's g (95% CI)	P_A_	Q_B_	P_B_
**Disease type**										
• Bipolar I	3	0.198 (0.094, 0.416)	< 0.001	7.889	0.005	2	0.562 (-0.663, 1.786)	0.368	0.018	0.893
• Bipolar I/II	7	0.755 (0.428, 1.333)	0.333			5	0.474 (0.062, 0.885)	0.024		
**Therapist background**										
• Psychologists	9	0.465 (0.245, 0.884)	0.020	1.411	0.235	4	0.448 (-0.191, 1.088)	0.169	0.014	0.906
• Nonpsychologists	1	1.200 (0.288, 4.993)	0.802			3	0.495 (0.074, 0.916)	0.021		
**Therapy delivery**										
• Individual	8	0.510 (0.265, 0.983)	0.044	0.000	0.997	6	0.373 (0.027, 0.719)	0.035	3.440	0.064
• Group	2	0.508 (0.067, 3.870)	0.513			1	1.348 (0.377, 2.319)	0.006		
**Treatment sessions**										
• ≥12	9	0.555 (0.298, 1.033)	0.063	1.850	0.174	5	0.466 (0.063, 0.869)	0.023	0.014	0.905
• <12	1	0.205 (0.056, 0.746)	0.016			2	0.542 (-0.638, 1.723)	0.368		
**Treatment frequency**										
• Otherwise	5	0.415 (0.141, 1.221)	0.110	0.243	0.622	3	0.355 (-0.128, 0.838)	0.150	0.285	0.593
• Weekly	5	0.578 (0.273, 1.222)	0.151			4	0.562 (-0.024, 1.148)	0.060		
**Treatment duration**										
• ≥90 min	2	0.508 (0.067, 3.870)	0.513	0.005	0.946	1	1.291 (0.361, 2.221)	0.006	3.004	0.083
• <90 min	4	0.548 (0.231, 1.300)	0.172			3	0.335(-0.218, 0.887)	0.235		
• undisclosed	4					3				

P_A_, subgroup effect on outcome variable; P_B_, heterogeneity among subgroups (moderator); CI, confidence interval

## Discussion

In the current study, we systematically reviewed the results of 19 RCTs and compared the treatment outcomes obtained by using CBT as an adjuvant therapy to pharmacotherapy and those obtained by using standard care for treating patients with BD. The research quality of the selected studies, including the quality of the study design, patients, outcome measures, statistical analysis, and results, was assessed using the approach described by Brodaty, Green, and Koschera [44]. According to the guidelines of the Cochrane Collaboration, a research quality score of 6–10 is acceptable. The 19 RCTs that received a total research quality score of >6 were included in the meta-analysis.

The meta-analysis indicated that CBT has a positive impact on patients with BD in terms of reducing depression levels, improving mania severity, decreasing relapse rates and increasing psychosocial functioning, with a moderate effect size. Our findings were similar to those of Jan [20] and Lam [21]. Compared to previous meta-analyses [17–19], we considered a greater number of databases and identified more RCTs that included four outcome measures (depression, mania, relapse rate, and psychosocial functioning) in the meta-analysis. In addition, we performed subgroup analyses of various characteristics, including disease type, therapists background, and treatment characteristics (such as therapy delivery type and session frequency and duration). Taken together, this meta-analysis derived more insights than previous studies through a comprehensive search and sophisticated analytic approaches.

Similar to that in unipolar patients, the underlying hypothesis for CBT application in BD is that these patients have distorted cognitions, which might lead to negative mood states. Nevertheless, CBT for BD also deals with distorted cognitions during manic states, termed “hyperpositive thinking,” which was not a treatment target in conventional CBT for patients with depression [45]. Although the role of regular treatment of BD episodes with antidepressants has yet to be established, the impressive results obtained for the use of CBT as an acute phase therapy for BD episodes suggest a critical avenue for future studies. Our findings suggest that CBT demonstrated greater effectiveness for reducing the relapse rate in patients with BD I compared with that in patients with BD I and II. This might be due to the relative homogeneity of the treatment population within these studies [25, 27, 37]. One possible explanation is the difference in disease course between BD I and BD II. The relapse rate for major depression tends to be higher in BD II than in BD I. In particular, determining the efficacy (or effectiveness) of CBT in real-world practice—both alone and as an adjuvant to monotherapy—for patients with BD II, in whom pharmaceutical therapy with mood stabilizers have unclear benefits, moreover, only one second-generation antipsychotic drug, quetiapine, has received Food and Drug Administration approval [14].

Our subgroup analysis revealed that treatment durations of ≥90 min per session were much more effective than were shorter treatment durations, and this treatment duration led to significantly improved depressive symptoms or mania severity. Previous studies on CBT with intervention durations ranging from 45 to 120 min per session have reported different extents of reduction in depression or mania levels [25–43], implying that the treatment duration is a potential moderator for treatment efficacy. Because CBT is a form of psychotherapy, it relies on a strong collaborative relationship between therapists and patients; this connection is strengthened by a more thorough process and longer treatment duration. In the future, treatment durations of ≥90 min might be implemented to increase effectiveness.

Among the 19 selected RCTs, patients with refractory BD were reported in two studies [39,43]. In the meta-analysis for determining the effect of CBT treatment on reducing depression and mania levels, the findings suggested that CBT had an impressive effect in patients with refractory BD [39]. Clearly, pharmacotherapy is an absolute necessity in this clinical syndrome, although this is not sufficient, at least in treatment-resistant patients. Combined CBT and pharmacotherapy might be an effective treatment strategy among patients with refractory BD.

Similar to most meta-analysis studies, the current study has some limitations. First, some comparisons were limited by the sample size. Only four studies [27, 29, 30, 38] had more than 100 patients, and the other RCTs involved small samples. Second, moderate-to-high heterogeneity was observed in the overall and subgroup analyses, indicating that a certain set of confounders (or possible personal and psychosocial factors), such as age, gender, and CBT style/approach, might be one of the heterogeneity sources affecting the results. Third, an important concern in meta-analyses is the “file-drawer” problem. Nonsignificant findings might not have been published, thus biasing the present results in a favorable direction for CBT. Although we conceive that this is not the case here (for example, the largest published RCTs of CBT in BD [29] had null findings), we calculated the number of studies with an effect size of zero that would be needed to reduce the present effect size to zero [46]. For the four different outcomes, depressive level, mania severity, relapse rate, and psychosocial functioning, 58, 43, 28, and 28 studies with no effect, respectively, would be needed to reduce the observed effect size to zero. These numbers are unlikely, considering that many of the published studies reported nonsignificant results. Collectively, more RCTs with larger sample sizes are warranted in the future to overcome these limitations, and the optimized and systematic approaches of CBT should be further investigated to prevent the effect of these factors in future studies. In addition, international, multicenter studies of a BD cohort with CBT might be valuable in establishing a database for the long-term evaluation of patient outcomes to facilitate evidence-based practices [47].

In conclusion, this meta-analysis recommends the use of CBT as an adjunctive therapy to medications in patients with BD because of the positive effects observed post-treatment and at follow-up. The benefits include decreased levels of depression and mania, decreased relapse rates, and increased levels of psychosocial functioning. The subgroup analysis indicated that the improvement in depression or mania levels was more profound with a CBT treatment duration of ≥ 90 min per session, and the relapse rate was lower among patients with BD I. Additional studies should investigate optimal patient selection strategies to maximize the benefits of adjunctive CBT and thereby the cost-effectiveness of treatment for patients with BD who do not rapidly respond to first-line interventions.

## Supporting information

S1 Fig Sensitivity analysis. Sensitivity analysis with leave-one-out approach of meta-analysis for (a) relapse rate, (b) level of depression, (c) severity of mania, and (d) level of psychosocial functioning of bipolar disorder among patients treated with CBT compared to control group.
(DOCX)

S2 Fig Funnel plot. Funnel plot for evaluating publication bias of meta-analysis for (a) relapse rate (Egger’s test: t = 1.81, df = 8, P-value = 0.107), (b) level of depression (Egger’s test: t = 2.83, df = 11, P-value = 0.016), (c) severity of mania (Egger’s test: t = 3.86, df = 9, P-value = 0.004), and (d) psychosocial functioning (Egger’s test: t = 2.08, df = 5, P-value = 0.092) of bipolar disorder among patients treated with CBT compared to control group.
(DOCX)

S1 Table (DOCX)

S1 PRISMA Checklist (DOC)
