# Supplementary material for: Efficacy of cognitive-behavioral therapy in patients with bipolar disorder: A meta-analysis of randomized controlled trials
Source: PLoS One. 2017 May 4;12(5):e0176849. doi: 10.1371/journal.pone.0176849 (PMC5417606; doi:10.1371/journal.pone.0176849)
Supplement: S1 Fig — Sensitivity analysis with leave-one-out approach of meta-analysis for (a) relapse rate, (b) level of depression, (c) severity of mania, and (d) level of psychosocial functioning of bipolar disorder among patients treated with CBT compared to control group. (DOCX) [file pone.0176849.s001.docx]

| **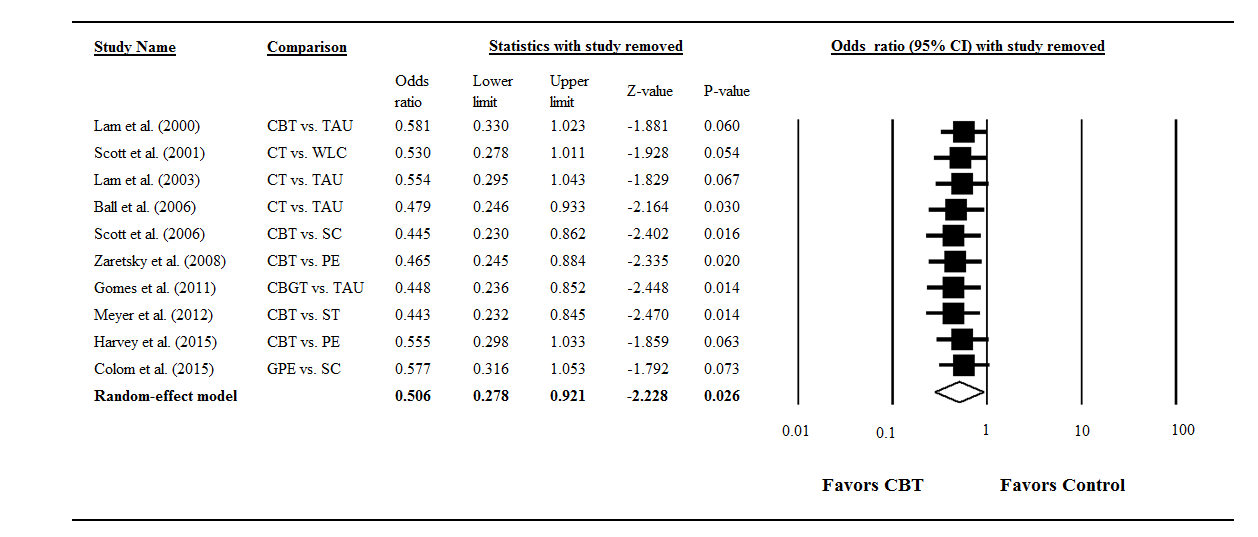(a)** |
| --- |
| **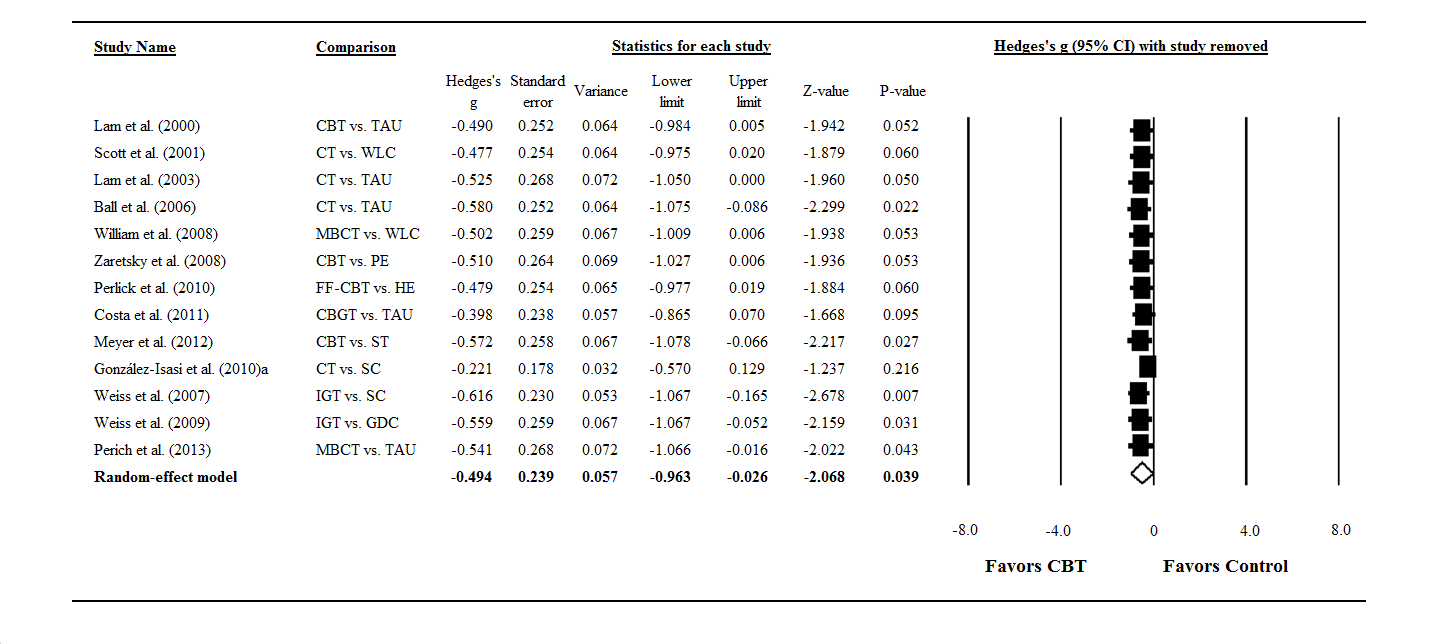(b)** |
|  |
| **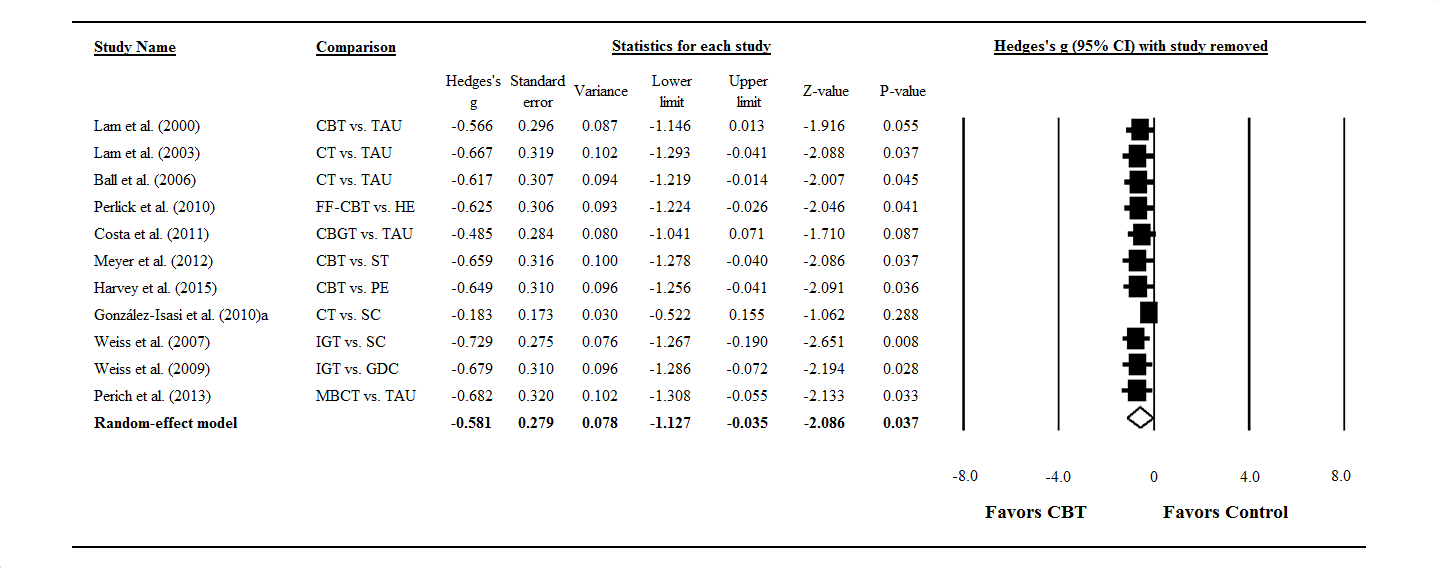(c)** |
| **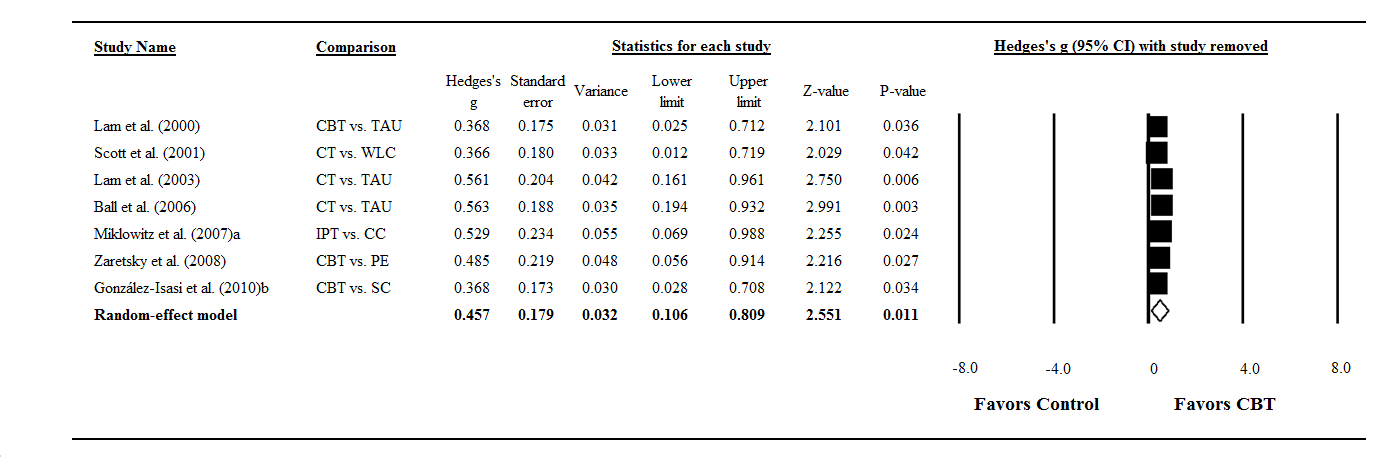(d)** |

**S1 Fig.** Sensitivity analysis with leave-one-out approach of meta-analysis for (a) relapse rate, (b) level of depression, (c) severity of mania, and (d) level of psychosocial functioning of bipolar disorder among patients treated with CBT compared to control group
