# Supplementary material for: Efficacy of cognitive-behavioral therapy in patients with bipolar disorder: A meta-analysis of randomized controlled trials
Source: PLoS One. 2017 May 4;12(5):e0176849. doi: 10.1371/journal.pone.0176849 (PMC5417606; doi:10.1371/journal.pone.0176849)
Supplement: S2 Fig — Funnel plot for evaluating publication bias of meta-analysis for (a) relapse rate (Egger’s test: t = 1.81, df = 8, P-value = 0.107), (b) level of depression (Egger’s test: t = 2.83, df = 11, P-value = 0.016), (c) severity of mania (Egger’s test: t = 3.86, df = 9, P-value = 0.004), and (d) psychosocial functioning (Egger’s test: t = 2.08, df = 5, P-value = 0.092) of bipolar disorder among patients treated with CBT compared to control group. (DOCX) [file pone.0176849.s002.docx]

| **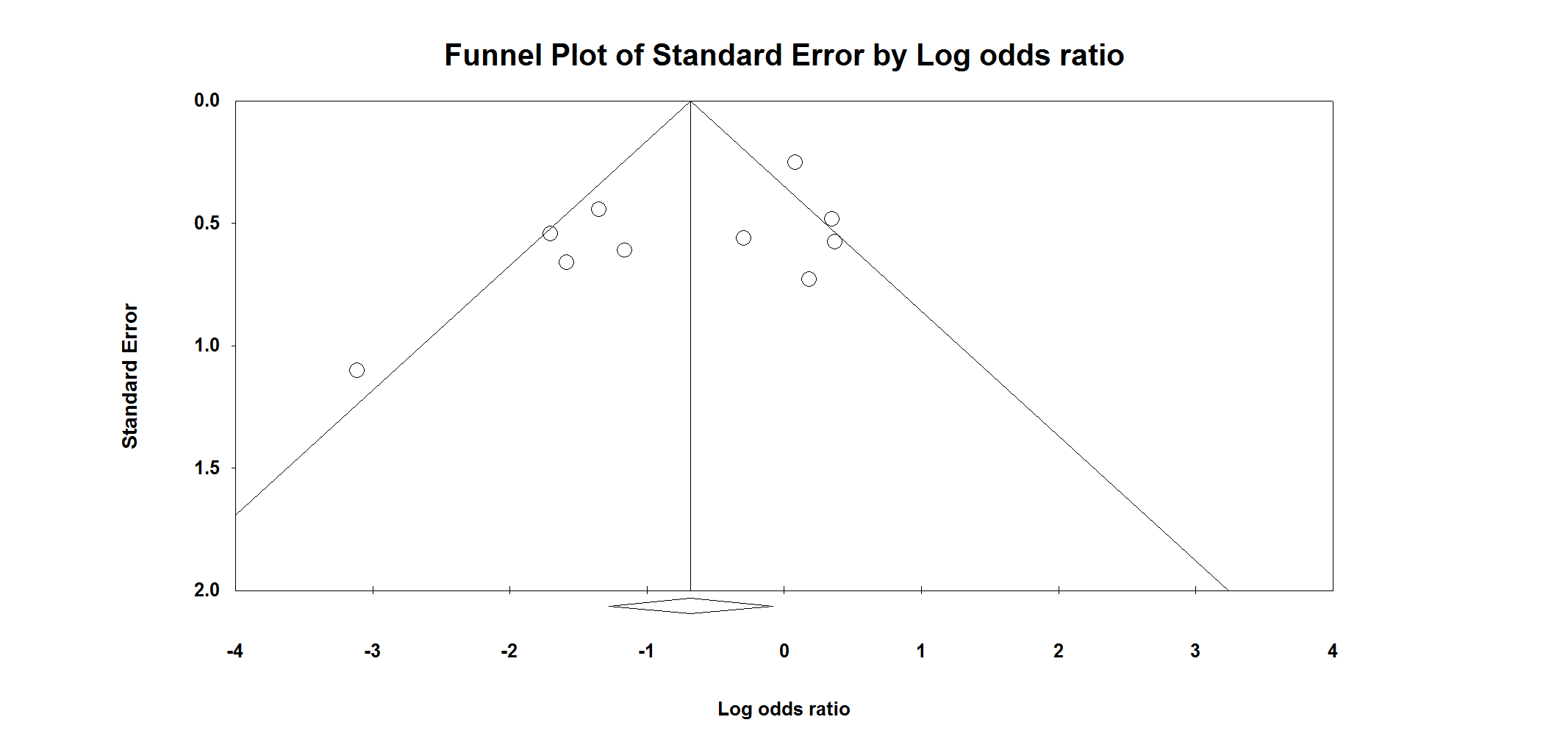(a)** |
| --- |
| **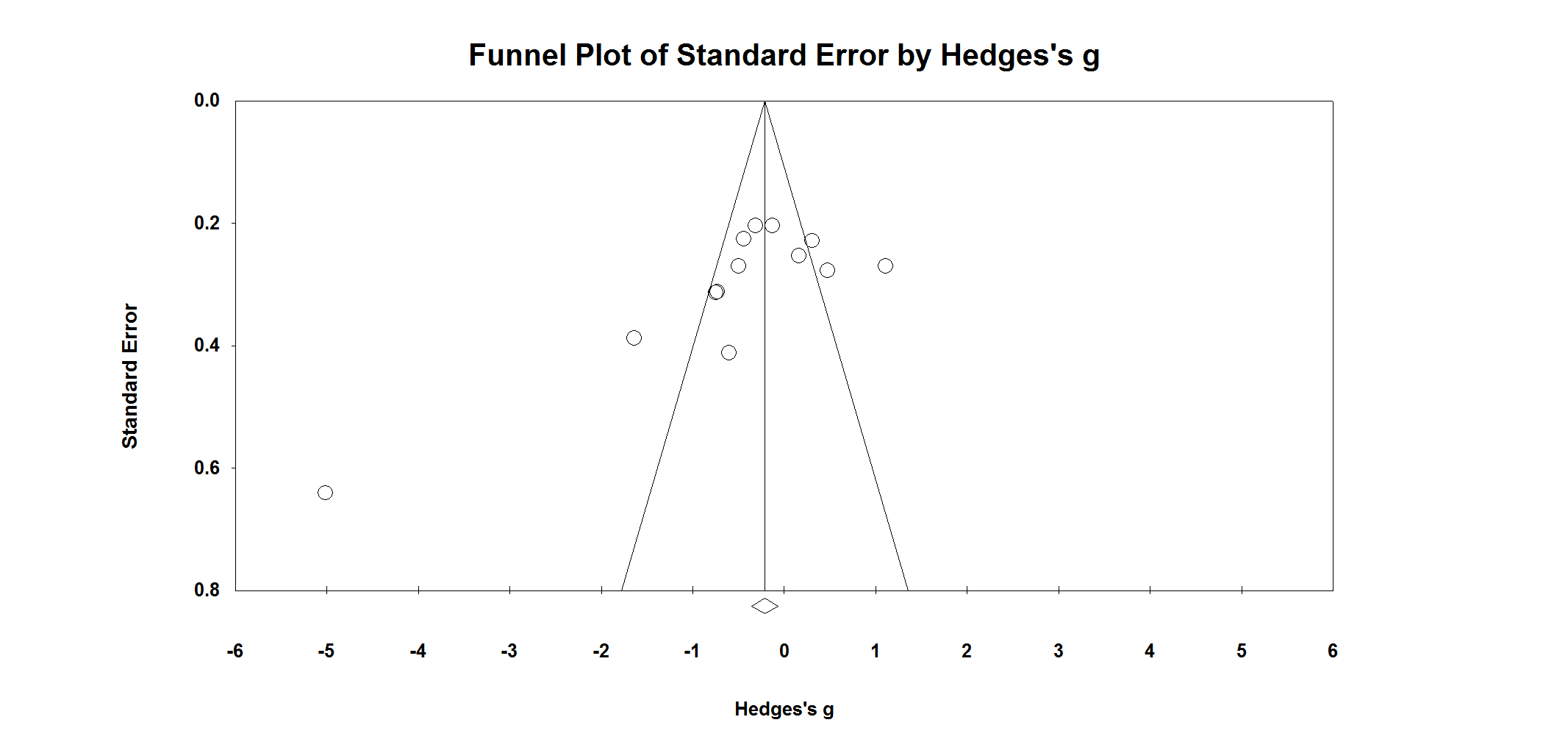(b)** |
| **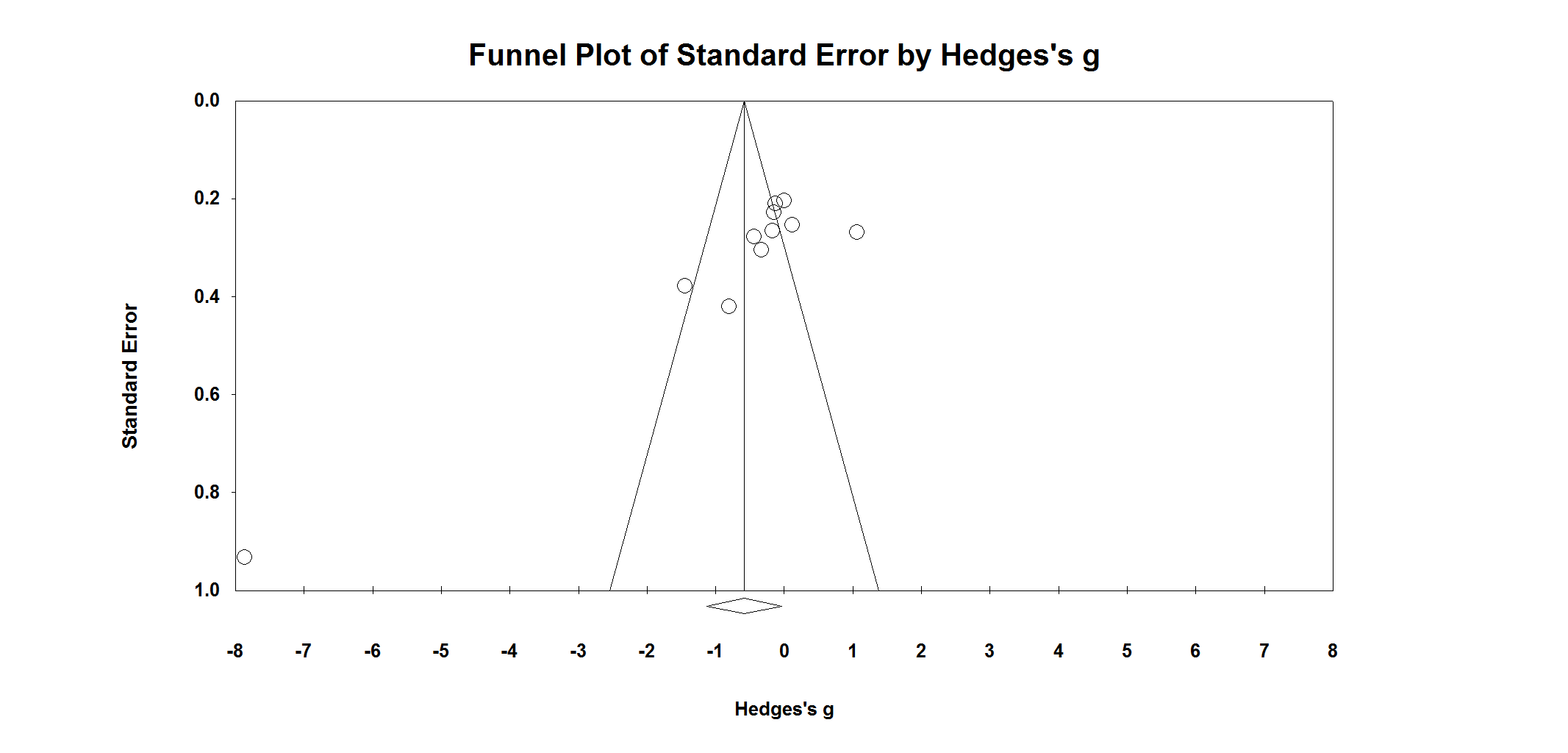(c)** |
| **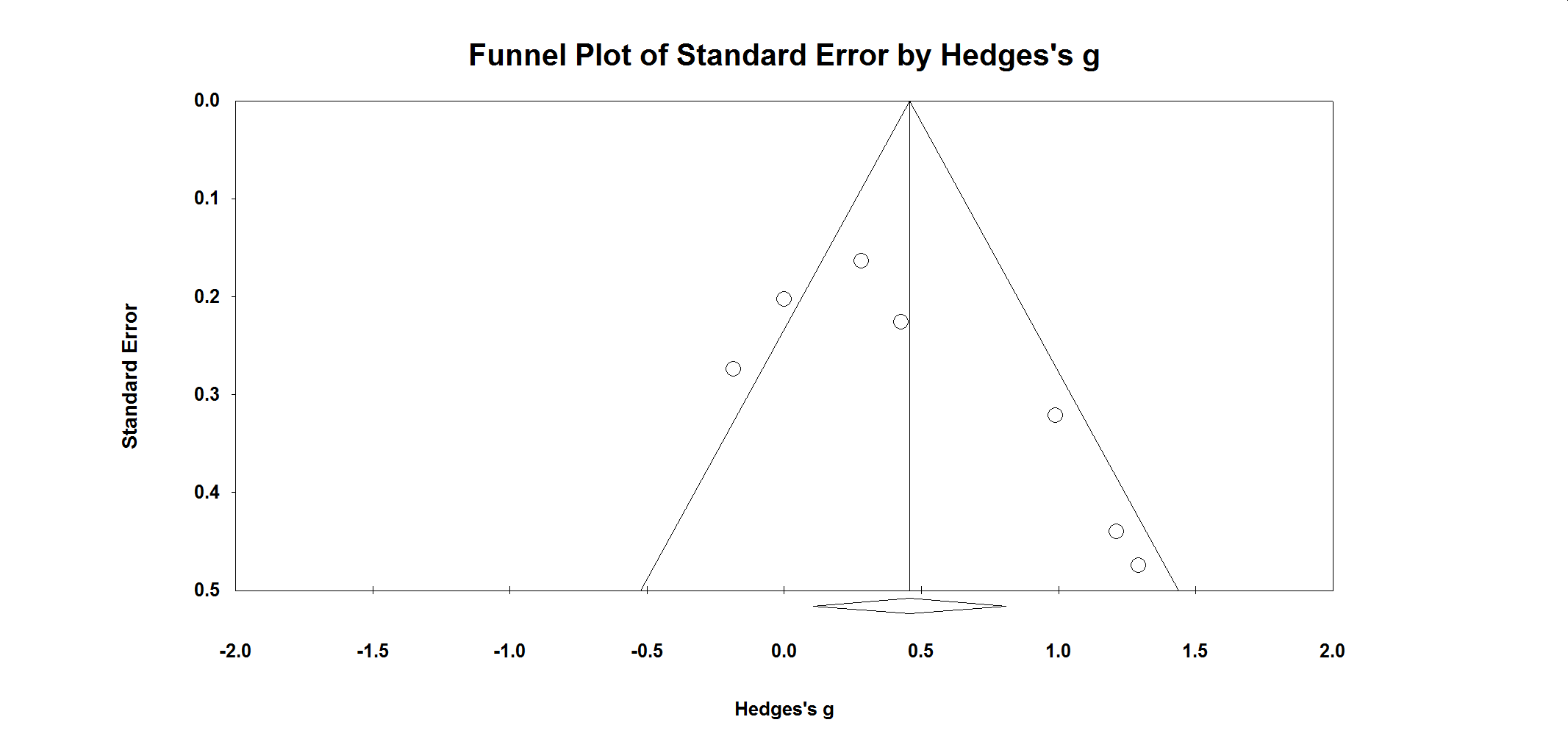(d)** |

**S2 Fig.** Funnel plot for evaluating publication bias of meta-analysis for (a) relapse rate (Egger’s test: t = 1.81, df = 8, P-value = 0.107), (b) level of depression (Egger’s test: t = 2.83, df = 11, P-value = 0.016), (c) severity of mania (Egger’s test: t = 3.86, df = 9, P-value = 0.004), and (d) psychosocial functioning (Egger’s test: t = 2.08, df = 5, P-value = 0.092) of bipolar disorder among patients treated with CBT compared to control group.
