# Supplementary material for: Efficacy of cognitive-behavioral therapy in patients with bipolar disorder: A meta-analysis of randomized controlled trials
Source: PLoS One. 2017 May 4;12(5):e0176849. doi: 10.1371/journal.pone.0176849 (PMC5417606; doi:10.1371/journal.pone.0176849)
Supplement: S1 Table — (DOCX) [file pone.0176849.s003.docx]

**Supplementary material**

S1 Table. Example for strategy of PubMed Search

| Search number | Search Terms |
| --- | --- |
| #1 | "Bipolar Disorder"[Mesh] OR "Bipolar Disorder"[Text Word] OR "manic-depressive psychosis"[Text Word] OR "bipolar affective disorder"[Text Word] OR "bipolar depression"[Text Word] |
| #2 | "Cognitive Therapy"[Mesh] OR "Cognitive-Behavioral Therapy"[Text Word] OR "psychotherapy"[Text Word] |
| #3 | #1 AND #2 |
| #4 | "Clinical Trial" [Publication Type] NOT (("Clinical Trial, phase I" [Publication Type]) OR ("Clinical Trial, phase II" [Publication Type])) |
| #5 | "Comment"[Publication Type] OR "Letter"[Publication Type] OR "Editorial"[Publication Type] OR "Review"[Publication Type] |
| #6 | (#3 AND #4) NOT #5 |
